# Estimating Z-ring radius and contraction in dividing *Escherichia coli*

**DOI:** 10.1111/j.1365-2958.2010.07087.x

**Published:** 2010-02-25

**Authors:** Johan Strömqvist, Karl Skoog, Daniel O Daley, Jerker Widengren, Gunnar von Heijne

**Affiliations:** 1 Department of Applied Physics, Royal Institute of TechnologySE-100 44, Stockholm, Sweden; 2 Center for Biomembrane Research, Department of Biochemistry and Biophysics, Stockholm UniversitySE-106 91 Stockholm, Sweden

## Abstract

We present a fluorescence recovery after photobleaching-based method for monitoring the progression of septal Z-ring contraction in dividing *Escherichia coli* cells. In a large number of cells undergoing division, we irreversibly bleached cytosolically expressed Enhanced Green Fluorescent Protein on one side of the septal invagination and followed the fluorescence relaxation on both sides of the septum. Since the relaxation time depends on the cross-sectional area of the septum, it can be used to determine the septal radius *r*. Assuming that the fraction of the observed cells with *r*-values in a given interval reflects the duration of that interval in the division process we could derive an approximate time-course for the contraction event, as a population average. By applying the method repeatedly on individual cells, the contraction process was also followed in real time. On a population average level, our data are best described by a linear contraction process in time. However, on the single cell level the contraction processes display a complex behaviour, with varying levels of activity. The proposed approach provides a simple yet versatile method for studying Z-ring contraction *in vivo*, and will help to elucidate its underlying mechanisms.

## Introduction

In *Escherichia coli*, cell division is initiated by the localization of FtsZ at the midcell [reviewed in (Weiss, 2004; Margolin, 2005; Lutkenhaus, 2007)]. Once localized, FtsZ polymerizes into a dynamic structure called the Z-ring. This ring can contract in the absence of other proteins (Osawa *et al.*, 2008) and is characterized by a high turnover of FtsZ molecules (Stricker *et al.*, 2002; Anderson *et al.*, 2004). Although the molecular structure of FtsZ is known (Lowe and Amos, 1998), the structure of the Z-ring is not. *In vitro* experiments indicate that purified FtsZ self-assembles into proto-filaments in the presence of GTP (Mukherjee and Lutkenhaus, 1994; Erickson *et al.*, 1996; Lowe and Amos, 1999). These protofilaments have also been observed *in vivo* using cryo-tomography reconstructions on *Caulobacter crescentus* cells (Li *et al.*, 2007), and are likely to be a basic building block of the Z-ring.

For division to proceed, approximately 10 other proteins must also be recruited to the midcell (Goehring and Beckwith, 2005). These proteins bring about the step-wise division of the cell, by tethering the Z-ring to the inner membrane, assisting in chromosome segregation and remodelling the peptidoglycan layer. Throughout the division process the Z-ring is thought to remain at the leading edge of the division site, as it contracts with the inner membrane.

Owing to the fact that FtsZ is widely conserved in bacteria (Vaughan *et al.*, 2004) and essential for cell division (Dai and Lutkenhaus, 1991), it is a target for antibiotic development (Haydon *et al.*, 2008; Lappchen *et al.*, 2008; Lock and Harry, 2008). As a consequence, there is interest in understanding how FtsZ assembles into the Z-ring and how the Z-ring contracts. Unfortunately, adequate tools are not available that can combine a sufficient spatial and temporal resolution with the possibility to study this dynamic process *in vivo*. Electron microscopy (EM), in particular cryo-EM tomography, provides a sufficient spatial resolution to view the arrangement of protofilaments in the Z-ring of certain bacteria (Li *et al.*, 2007). However, the technique is highly destructive and difficult to combine with functional assessments *in vivo* of the cell division process. Moreover, high-resolution cryo-EM cannot be applied to thicker cells, such as *E. coli*. In contrast, fluorescence spectroscopy is compatible with live cell functional imaging, and can offer a wealth of information. In the last few years, fluorescence-based microscopy techniques have also been developed that can offer a spatial resolution well beyond the diffraction limit of resolution (see Hell, 2007 for a review). Nonetheless, the spatial resolution is still too low to monitor the full range of dimensions of the septal region of an *E. coli* cell undergoing division.

In this study we have utilized a novel approach to allow high resolution assessment of the progressing Z-ring contraction in dividing *E. coli* cells. Fluorescence recovery after photobleaching (FRAP) measurements of cytosolic Enhanced Green Fluorescent Protein (EGFP) were performed on a large number of *E. coli* cells at different stages of cellular division. In the FRAP experiments the EGFP content was irreversibly bleached in one of the two compartments, and the relaxation time towards a uniform EGFP concentration over both compartments was measured. As the relaxation time depends on the cross-section of the invaginating septum, it can be used to estimate the radius of the septal region. Modelling the septal region as a cylindrical channel with a variable radius we derive a relation between the relaxation time and the septal radius. The resulting distribution of radii, representing a pseudo time-course of the division process as a population average, was complemented by single cell data from repetitive measurements on individual dividing cells.

## Results and discussion

### FRAP measurement on dividing *E. coli* cells

In order to study the contraction of the Z-ring *in vivo*, we sought a method that would enable us to measure the diameter of the septal ring during the division event. We reasoned that we could calculate the diameter by irreversibly photobleaching EGFP on one side of the septal invagination and measuring the relaxation to a uniform fluorescence between the two sides caused by the diffusion of EGFP from the other half, assuming that the cross-sectional area of the Z-ring would control the relaxation time.

**Fig. 1:**
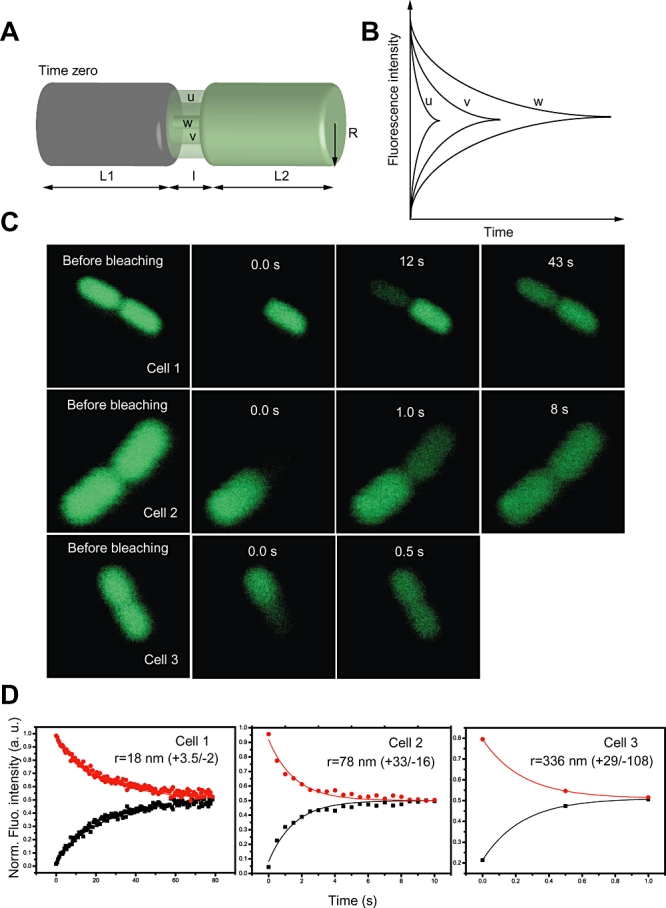
A. Schematic picture of a dividing cell with different septal radii (= u, v, w), where the EGFP content in one of the compartments is depleted by photobleaching, and where the equilibration in EGFP concentration over the compartments is then followed as a function of time after photobleaching. B. Corresponding fluorescence recovery curves. C. Consecutive images of three different dividing *E. coli* cells subject to a typical FRAP experiment. D. The bleach- and drift-corrected normalized EGFP fluorescence in the bleached (black) and non-bleached (red) cell volume versus time after bleaching, for the image sequences of the cells shown in panel C. The apparent ‘mirror image’ of the black and red curves is an effect of the normalization and does not appear in the raw data.

Exponentially growing *E. coli* cells expressing EGFP were immobilized in soft agar on a coverslip. 222 cells with a visible short invagination in the centre and with a length less than 7 µm were selected for investigation. Initially the cells were partially bleached in one of the ends (spot diameter 0.6 µm, bleach time 20–70 ms) to verify that EGFP diffusion was normal (i.e. no remaining concentration gradient of fluorescent EGFP in that cell compartment 0.8 s after the bleach period). Cells with a clearly reduced EGFP diffusion coefficient were excluded from further FRAP experiments (23% of all cells). The remaining cells were subject to FRAP experiments as illustrated in Fig. 1. In each of the cells, the entire cell volume on one side of the invagination was irreversibly bleached (Fig. 1A). The EGFP fluorescence was then followed over time in the central region of both the non-bleached and the bleached cell volume, as a recovery in the bleached compartment and a corresponding decay in the non-bleached compartment (Fig. 1B). Following photobleaching, the fluorescence within both compartments of the cell was scanned by the laser every 0.5 s. In Fig. 1C a set of consecutive images of three different dividing *E. coli* cells are shown to illustrate this procedure. For further analysis, the fluorescence intensity, in two equally sized circular regions (of diameter ∼0.3 µm) within the confocal scanning plane and in the centre of each compartment, was recorded at each time point. Correction for bleaching and drift during scanning was done by normalizing the intensity with the total weighted fluorescence *f*(*t*), as described in *Experimental procedures* (Eq. 4). Figure 1D shows bleach- and drift-corrected fluorescence data for the same cells as imaged in Fig. 1C.

### Estimation of the septal ring radius from the FRAP data

In order to estimate the septal ring radius *r* from the fluorescence recovery and decay curves, we derived a formula based on Fick's first law. In a geometry consisting of two compartments (volumes *V_1_* and *V_2_*) inter-connected by a cylindrical septal region of length *l* and radius *r*, the normalized average fluorescence intensity in each compartment is described by


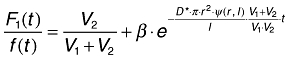
(1A)

and


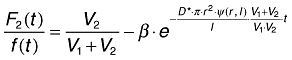
(1B)

Here, *F*_1_(*t*) and *F*_2_(*t*) denote the recorded fluorescence intensity in the volumes *V_1_* and *V_2_*, *β* is an arbitrary constant, and *ψ*(*r*,*l*) is a dimension-less correction factor accounting for the influence of the EGFP concentration gradient in the septal region. *ψ*(*r*,*l*) is generated from numerical simulations (described below). D* is the diffusion coefficient of EGFP in the septal region, taking steric and hydrodynamic hindrance into account. It is calculated from the cytosolic diffusion coefficient *D* as


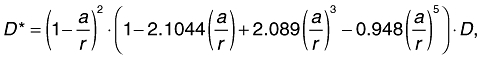
(2)

where the first factor describes steric and the second hydrodynamic hindrance (Renkin, 1954; Anderson and Quinn, 1974) and *a* is the average hydrodynamic radius of the EGFP molecule (= 2.4 nm) (Ormö*et al.*, 1996). The second factor, the so-called centerline approximation, is valid when *a*/*r* < 0.4, i.e. when *r* > 6 nm.

**Fig. 2:**
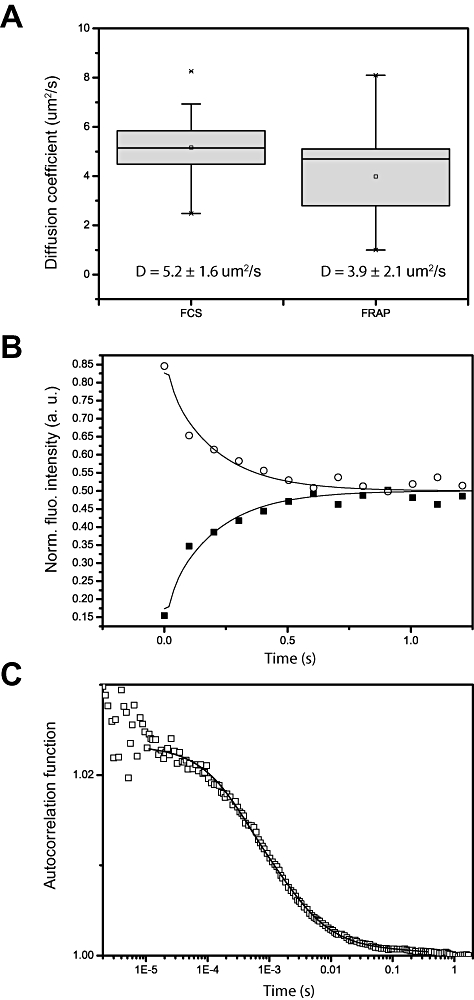
A. A boxplot of the diffusion coefficients derived from FRAP (left box) and FCS (right box) experiments on elongated cells lacking a visible septum (13 and 12 cells respectively). B. A typical normalized FRAP curve, where filled squares indicates the recovery in the centre of the bleached half and non-filled circles the decay in the non-bleached half versus time after bleaching. Black lines are the corresponding fits according to Eq. S5A and B. C. A typical autocorrelation curve (non-filled squares) with a fit (black line) according to the two-component model in Eq. S6.

The diffusion coefficient *D* itself was independently determined by FRAP and Fluorescence Correlation Spectroscopy (FCS) within the *E. coli* cytoplasm of a set of elongated cells lacking a visible septum (Fig. 2A). Both FRAP and FCS yielded *D* ≈ 4.5 ± 1.5 µm^2^ s^−1^, which is comparable with earlier studies by FRAP (Elowitz *et al.*, 1999; Mullineaux *et al.*, 2006) and FCS (Meacci *et al.*, 2006). Examples of typical FRAP and FCS curves are shown in Fig. 2B and C. The models used to determine *D* by FRAP and FCS are described in detail in the Supporting Information. The diffusion properties of EGFP in the cells analysed were never found to be significantly different in the two separated compartments within an individual dividing cell.

To refine the analysis of the fluorescence recovery data Finite Element Method (FEM) simulations (Strang and Fix, 1973) were performed. In the simulations, the EGFP concentration, obeying the three-dimensional diffusion equation, was followed in time, within a geometry consisting of two compartments of cylindrical shape (Fig. 1A), after full bleaching of one of the compartments for 40 ms. The two compartments of radius *R* = *500*nm and lengths *L*_1_ = *L*_2_ = 2 µm were inter-connected by a septal cylindrical region of length *l*. *l* was specified to 50 nm in the simulations, based on literature values determined by EM (Burdett and Murray, 1974). Effects from steric and hydrodynamic hindrance on the diffusion coefficient, according to Eq. 2, were also incorporated into the model. By simulating the FRAP experiment in this manner, relaxation curves were generated for different radii. These relaxation curves were then fitted to Eq. 1A and B. In the fits, all the parameters were fixed to the same values as used as input values in the simulations, except *ψ*(*r*,*l*) and *β* which were allowed to vary freely as fitting parameters. Thereby, a relation between *r* and *ψ*(*r*,*l*), with *D* = 4.5 µm^2^ s^−1^ and *r* from 6 to 500 nm, was empirically found (see Supporting Information, Eq. S14).

Within the context of this analysis, Eqs. 1A and B are valid for any reasonable diffusion coefficient. Both cylindrical and ellipsoid compartments are allowed. The septal radius, *r*, should be between 6 nm and approximately 80% of *R*, the radius of the compartments. When *r* approaches 6 nm, steric and hydrodynamic hindrance becomes too dominant, see Eq. 2. When the radius is comparable with *R*, then mainly differences in *D* between cells affect the relaxation time, and not the septal invagination. If *R* = 500 nm then radii larger than ∼400 nm are not possible to distinguish from each other with this method.

### Cumulative distribution of *r*

**Fig. 3:**
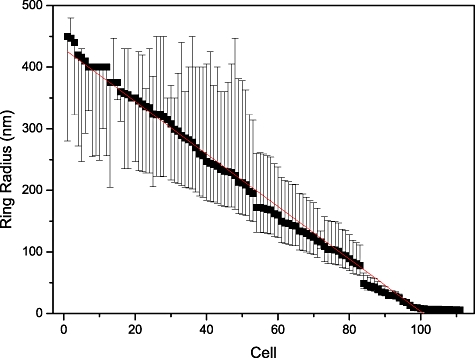
Distribution of estimated ring radii *r* for 111 cells. Error estimates based on the uncertainty of the diffusion coefficient, *D*, indicated by bars. The red line represents a linear fit to the *r*-distribution, yielding an intercept and slope of 430 nm and −4.3 nm/measurement respectively.

The normalized experimental relaxation curves could in general be fitted nicely to Eq. 1A and B, as demonstrated in Fig. 1D. Thirty-five per cent of the cells investigated were found to have *r* < 6 nm. Among these cells, only 10% showed some tendency to recover fluorescence within 1 min in the FRAP experiment. Cells with smaller radii than 6 nm were not included in the further analysis, as the effects of steric and hydrodynamic hindrance of the EGFP molecules are increased and difficult to predict. We nevertheless note that the fairly large fraction of cells with *r* < 6 nm suggests that the final closing of the septum and separation of the daughter cells account for a substantial part of the entire time-course of the division process. The observed radii of the remaining 111 cells were sorted into a histogram, Fig. 3. The error bars in Fig. 3 result from the standard deviation in the cytosolic diffusion coefficient *D* (see above).

Given that the FRAP measurements were performed on the 111 cells at random stages of cellular division (but after a visible septal invagination had formed), the number of cells that are measured at a particular stage of division is proportional to the duration of that stage within the time of the division process. The distribution of *r* among the 111 cells thus reflects the population-average time development of the septal contraction from the time when a septum is visible until *r* has been reduced to ∼6 nm. This distribution is well described by a linear function (Fig. 3, red line). According to Fig. 3, the largest radii measured were approximately 430 nm. Only cells for which an invagination could be observed were subject to analysis. However, an invagination may be difficult to detect by visual inspection at an early phase. Therefore the population of cells with *r* > 400 nm might be underestimated.

As a negative control, we performed the same fitting on the cells lacking a visible septum, which were used to determine the diffusion coefficient of EGFP. The resulting distribution consisted only of radii larger than 400 nm (data not shown).

### Measurement of radial contraction on individual cells

To study the temporal dependence of *r* directly, we performed consecutive FRAP measurements on individual cells. In most cells there was enough EGFP to sustain up to three rounds of bleaching/recovery while maintaining a sufficient amount of non-bleached EGFP to be able to estimate *r*. Partial bleach measurements were performed before and after all relaxation measurements to assure that *D* did not change significantly.

**Fig. 4:**
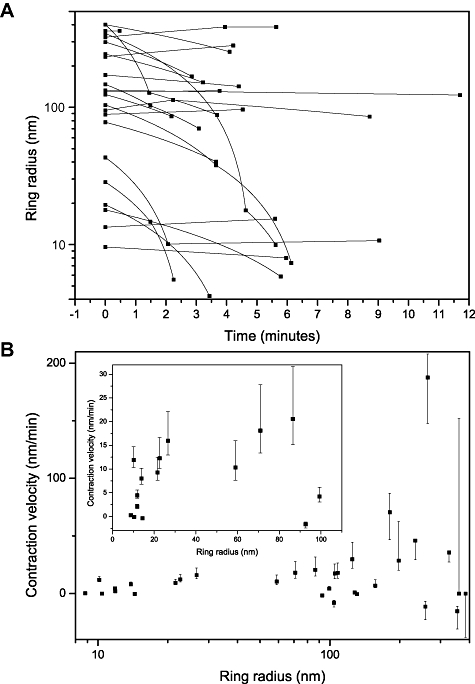
A. Measured ring radii versus time obtained from consecutive bleaching experiments on 23 cells. The black lines illustrate the radial time dependence assuming a linear contraction in between two separate measurements. B. Corresponding estimated radial contraction velocities for these 23 cells, versus estimated septal ring radius at time of measurement. Inset: Magnified plot of contraction velocity versus septal ring radius for all cells with *r* < 100 nm. Error estimates based on the uncertainty of the diffusion coefficient, *D*, indicated by bars.

Twenty-three cells were analysed individually at two or three successive times, Fig. 4. Their individual contraction velocities were estimated as 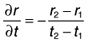, where *r*_1_ and *r*_2_ are the measured septal radii at times *t_1_* and *t_2_*.

In particular, some cells having septal radius larger than ∼80 nm were found to contract with much higher velocity than cells with smaller septal radii. In a few cases the contraction velocity was close to or even exceeded 50 nm min^−1^. The variation of the velocities was large, not only between cells but also within the same cell during different measurements, and a relatively large fraction of the cells showed no contraction at all. In Fig. 4 it appears as if some of the cells would have slightly negative velocity. However, these contraction velocities, 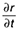, are all within the error margins of being zero. Regarding the negative contraction velocities as zero and excluding a single cell for which the contraction velocity was close to 200 nm min^−1^, yields an average contraction velocity of 12 ± 16 nm min^−1^. This average contraction velocity seems to remain constant over the full range of radii, in agreement with the ensemble data of Fig. 3.

Recently, Reshes *et al.* (2008) proposed a model for the septal contraction dynamics of dividing *E. coli* cells based on phase-contrast imaging data. In the model, the relative contraction of the septal radii is proportional to 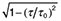, where *τ* is the time after initiation of constriction and *τ*_0_ the total time of contraction (Reshes *et al.*, 2008). This model does not agree well with the averaged linear contraction found in this work (Fig. 3). However, their model is based on data acquired in the initial phase of contraction only (*r* > 300 nm), while our data mainly characterizes the rest of the contraction process. The proposed model of Reshes *et al.* (2008) is therefore not necessarily in contradiction to our results. From our data, given a linear contraction process and the average contraction velocity determined in Fig. 4, a full contraction would last for 35 min. This is somewhat longer than the 7 and 19 min contraction times that have previously been reported for *E. coli* at 37°C in rich and minimal liquid media respectively (Sun and Margolin, 1998; Reshes *et al.*, 2008). However, the FRAP measurements presented in this work were performed on cells expressing EGFP in a semi-solid medium at room temperature. The contraction times derived here are thus reasonable, in view of those found in the previous investigations.

### Conclusions

The approach established in this work is simple, yet it enables a high-resolution assessment of the contraction of the Z-ring in dividing *E. coli* cells. The methodology relies critically on an objective selection of the cells to be analysed. This requires well-defined selection criteria, which can be judged under the microscope by visual inspection. The selection process in this study (visible invagination, total length < 7 µm) meets these requirements, and should lend itself to an automated procedure.

Taken together, our results show that, as a population average, the contraction of the Z-ring can be approximated by a linear decrease in radius *r* with time in the interval 400 nm > *r* > 6 nm. However, at the single cell level the contraction process appears much more complex, as a mixture of processes of different speeds in combination with certain probabilities to halt. Altogether, these data place important constraints on attempts to model the cell-division process in *E. coli*. We hope that the approach will become a useful tool for further studies of cell division in *E. coli* and other single-cell organisms.

## Experimental procedures

### Strains and plasmids

The pET20b-EGFP-His_8_ plasmid (Drew *et al.*, 2006) was transformed into *E. coli* strain BL21(DE3). A colony was inoculated into a 2 ml tube containing 1 ml of LB media with ampicillin (50 µg ml^−1^), and incubated with vigorous shaking at 37°C for 16 h. The culture was back-diluted 1/20 and incubated as before for 3 h, until the OD_600_ was between 0.3 and 0.5. Cells were harvested by centrifugation at 830 *g* for 5 min and the cell pellet was washed three times in 1× PBS buffer. The final cell pellet was resuspended in 1 ml of 1× PBS buffer. Note that EGFP-His_8_ was produced by ‘leaky’ expression from the pET20b-EGFP-His_8_ plasmid (no inducer was added).

### Microscopy and photobleaching

Cells were placed on a cover glass slip, immobilized by adding soft agar (1%) and analysed within 1 h in room temperature. Fluorescence microscopy was performed on a Carl Zeiss ConfoCor 3 confocal laser scanning microscope using a C-Apochromat 40× objective with a numerical aperture of 1.2. The 488-line of a 30 mW argon ion laser was used for both photobleaching and the subsequent fluorescence excitation/recording. The radius of the laser in the focal plane was estimated to 0.2 µm. For photobleaching, a power of 1.4 mW was applied onto selected regions within one of the compartments of the cell for 40 ms. Following photobleaching, the fluorescence within the compartment exposed was nearly fully depleted. Thereafter, the cell was scanned with a laser excitation power of 5 µW every 0.5 s for the FRAP measurements made to determine the radius, and every 0.1 s for the FRAP measurements made to determine the diffusion coefficient. In all FRAP analyses the fluorescence intensity was recorded in two equally sized circular regions (diameter of ∼0.3 µm) within the confocal scanning plane and in the centre of each compartment. The fluorescence was detected by two avalanche photodiodes after passage through a dichroic mirror (HFT 488/543/633), a pinhole in the image plane (edge-to-edge distance 300 µm) and an emission filter in front of each detector (BP 505–540 IR). For the FCS diffusion coefficient measurements, the same instrument was used, but with a pinhole of 70 µm and an emission filter BP 505–530 IR. The waist *ω*_0_ of the detection volume was determined to be 181 ± 10 nm from calibration measurements with Rhodamine110.

### Correction for drift and bleaching in the FRAP measurements

In the monitoring phase, following the photobleaching pulse and during scanning, the relative influence of bleaching and drift on the recorded fluorescence intensity are described by the functions *B*(*t*) and *M*(*t*) respectively. The fluorescence intensity *F_i_*(*t*) in compartment *i* can then be denoted:


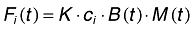
(3A)

where *K* is a proportionality constant. Hence:


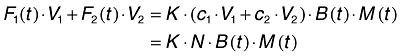
(3B)

where *N* is the constant number of fluorophores in the cell immediately after the photobleaching pulse. By defining normalized total fluorescence intensity *f*(*t*) as:


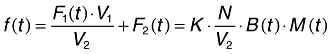
(4)

it is possible to get rid of bleaching and drift artefacts by dividing *F_i_*(*t*) by *f*(*t*):


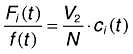
(5)

The drift- and bleaching-corrected measurements from both compartments were then fitted according to Eq. 1A and B. The compartment lengths were estimated by visual inspection of the confocal images before photobleaching.

### Curve fitting and numerical FEM simulations

Curve fitting was performed using the Origin 8 software (OriginLab Corporation, Northampton). The numerical simulations based on the finite element method were made using the FEM program package Comsol Multiphysics 3.5. The geometry was divided into ∼10 000 elements and the time steps were chosen freely by the solver.
