# Supplementary material for: Estimating Z-ring radius and contraction in dividing Escherichia coli
Source: Mol Microbiol. 2010 Feb 25;76(1):151–8. doi: 10.1111/j.1365-2958.2010.07087.x (PMC2871166; doi:10.1111/j.1365-2958.2010.07087.x)
Supplement: Supplementary file 1 [file mmi0076-0151-SD1.pdf]

Supplementary Methods

Models used to determine the cytosolic diffusion coefficient of EGFP from FRAP and FCS measurements. The analysis of FRAP data on cells, with length L and without any septum, was based on the one-dimensional diffusion equation:

$$\frac{\partial c}{\partial t} = D \cdot \frac{\partial^2 c}{\partial x^2} \qquad \text{(S1)}$$

with boundary conditions:

$$\frac{\partial c(0,t)}{\partial x} = \frac{\partial c(L,t)}{\partial x} = 0 \qquad \text{(S2)}$$

The general solution to this boundary value problem is a Fourier series

$$c(x,t) = \sum_{n=0}^{\infty} a_n \cdot e^{-q_n^2 \cdot D \cdot t} \cdot \cos(q_n x)$$

where $q_n = \frac{n \cdot \pi}{L}$, $n = 0,1,2,...$ and each a_n is determined by the initial condition. Since the bleaching time is chosen relatively short compared to the diffusion of the EGFP molecules, the initial condition, after bleaching half of the cell, is approximately a step function from a constant low concentration, c_1, of fluorescent molecules in the bleached half to a constant higher concentration, c_2, in the unbleached half, i.e.

$$c(x,0) = \begin{cases} c_1, 0 < x < \frac{L}{2} \\ c_2, \frac{L}{2} < x < L \end{cases} \qquad \text{(S3)}$$

Eq. S1, with the boundary conditions of S2 and the initial condition of S3 yields the following solutions in the center of each half of the cell:

$$c(\frac{L}{4},t) = \frac{c_1 + c_2}{2} - \frac{\sqrt{2}}{\pi} \cdot (c_2 - c_1) \cdot (S_1(t) + S_3(t) - S_5(t) - S_7(t)) \qquad \text{(S4A)}$$

and

$$c(\frac{3}{4}L,t) = \frac{c_1 + c_2}{2} + \frac{\sqrt{2}}{\pi} \cdot (c_2 - c_1) \cdot (S_1(t) + S_3(t) - S_5(t) - S_7(t)) \qquad \text{(S4B)}$$

where $S_i(t) = \sum_{n=1}^{\infty} \frac{e^{-D\cdot(8\cdot(n-1)+i)^2\cdot\pi^2\cdot t/L^2}}{8\cdot(n-1)+i}$. The detected fluorescence is proportional to the concentration; hence the relative fluorescence intensity in the center of each half of the cell is given by:

$$\frac{F_1(t)}{F_1(t)+F_2(t)} = \frac{K*c(\frac{L}{4},t)}{K*c(\frac{L}{4},t)+K*c(\frac{3}{4}L,t)} = \frac{1}{2} - \frac{\sqrt{2}}{\pi}\cdot\frac{F_2(0)-F_1(0)}{F_2(0)+F_1(0)}\cdot(S_1(t)+S_3(t)-S_5(t)-S_7(t)) \quad \text{(S5a)}$$

and

$$\frac{F_2(t)}{F_1(t)+F_2(t)} = \frac{K*c(\frac{3}{4}L,t)}{K*c(\frac{L}{4},t)+K*c(\frac{3}{4}L,t)} = \frac{1}{2} + \frac{\sqrt{2}}{\pi}\cdot\frac{F_2(0)-F_1(0)}{F_2(0)+F_1(0)}\cdot(S_1(t)+S_3(t)-S_5(t)-S_7(t)) \quad \text{(S5B)}$$

where *K* is a proportionality constant and $F_1(t)$ and $F_2(t)$ describes the detected fluorescence in the center of the first and second half of the cell, respectively. In the experimental FRAP curves, the measured $F_1(t)$ and $F_2(t)$ were fitted simultaneously to Eq. S5A and S5B, respectively, including only the first two terms in each infinite sum $S_i(t)$ (since the magnitude of the remaining terms are negligible compared to the first two).

For the FCS measurements, the experimental autocorrelation curves were fitted to the following model:

$$G(\tau) = \frac{1}{(\mathrm{N}_1+\mathrm{N}_2)^2}\cdot\left(\frac{\mathrm{N}_1}{1+4\cdot\mathrm{D}_1\cdot\tau/\omega_0^{\,2}} + \frac{\mathrm{N}_2}{1+4\cdot\mathrm{D}_2\cdot\tau/\omega_0^{\,2}}\right)\cdot\frac{1-\mathrm{T}+\mathrm{T}\cdot e^{-\tau/\tau_\mathrm{T}}}{1-\mathrm{T}} + 1 \qquad \text{(S6)}$$

Here, N_1 and N_2 are the number of molecules, within the detection volume, with diffusion coefficients D_1 and D_2, respectively. T is the average fraction of molecules that temporarily resides in a dark state, and with a relaxation time τ_T related to the corresponding blinking (Widengren *et al.*, 1999, Haupts *et al.*, 1998). In the model, a two-dimensional diffusion of the molecules is assumed, since the height of the detection volume is larger than the diameter of the bacterium. In the fitting of Eq. S6 to the experimental FCS curves, τ_T was fixed to 300 μs. This is a typical relaxation time found in FCS measurements for GFP under the excitation conditions used (see Materials and Methods) and the expected pH of the *E. coli* cytoplasm

(pH ~ 7). The origin of this relaxation component was not investigated further, but may be either due to a photo-induced non- or weakly fluorescent state of EGFP, or follow from proton exchange. In the experimental FCS curves, the fraction of the faster component ($=\frac{N_1}{N_1+N_2}$) was 0.93 ± 0.03, indicating that most of the dynamics can be attributed to free diffusion of EGFP.

Model to determine the septal radius from FRAP curves The model is based on a geometry, consisting of two compartments with volumes V_1 and V_2, interconnected by a cylindrical septal region of length l and radius r. According to Fick's first law the net number of molecules n passing through the septum connecting the two compartments, satisfy:

$$\frac{\partial n}{\partial t} = A \cdot D \cdot \frac{\partial c}{\partial x} \qquad \text{(S7)}$$

Here, c is the concentration, $\frac{\partial c}{\partial x}$ is the concentration gradient along the symmetry axis, D is the diffusion coefficient of the molecules, and A is the cross section of the channel. If the molecules undergo diffusional exchange much faster in the two compartments than through the septal region, then the gradient $\frac{\partial c}{\partial x}$ (full line in Fig. S5A) can be approximated by $\frac{c_2 - c_1}{l}$ (dashed line in Fig. S5A), where c_i is the average concentration in compartment i. By introducing $\psi(r,l) = \frac{\partial c}{\partial x} / \frac{c_2 - c_1}{l}$, a function that corrects for the error in approximating the derivative by the overall concentration difference between the daughter cells, the gradient can be expressed as $\frac{c_2 - c_1}{l} \cdot \psi(r,l)$. Now, based on Eq. S7, the number of molecules entering compartment 1 per time unit is given by:

$$\frac{\partial N_1}{\partial t} = \frac{A \cdot D \cdot \psi(r,l)}{l} \cdot \left(\frac{N_2}{V_2} - \frac{N_1}{V_1} \right) = \frac{A \cdot D \cdot \psi(r,l)}{l} \cdot \left(\frac{N - N_1}{V_2} - \frac{N_1}{V_1} \right) =$$
$$= \frac{c_2 - c_1}{l} \frac{A \cdot D \cdot \psi(r,l)}{l} \cdot \left(\frac{N}{V_2} - N_1 \cdot \left(\frac{V_1 + V_2}{V_1 \cdot V_2} \right) \right) \qquad \text{(S8)}$$

where $N = N_1 + N_2$ is the constant total number of molecules, and N_i and V_i are the number of molecules and the volume of compartment i. The general solution to Eq. S5 is:

$$N_1 = N \cdot \frac{V_1}{V_1 + V_2} + \alpha \cdot e^{-\frac{D \cdot \pi \cdot r^2 \cdot \psi(r,l)}{l} \cdot \frac{V_1 + V_2}{V_1 \cdot V_2} \cdot t} \quad \text{(S9)}$$

where α is an arbitrary constant.

The fluorescence $F_i(t)$ is proportional to the concentration of fluorophores, N_i / V_i, hence:

$$\frac{F_1(t)}{f(t)} = \frac{N_1 / V_1}{N_1 / V_2 + (N - N_1) / V_2} = \frac{N_1 \cdot V_2 / V_1}{N} = \frac{V_2}{V_1 + V_2} + \beta \cdot e^{-\frac{D \cdot \pi \cdot r^2 \cdot \psi(r,l)}{l} \cdot \frac{V_1 + V_2}{V_1 \cdot V_2} \cdot t} \quad \text{(S10)}$$

where β is an arbitrarily constant and $f(t) = F_1(t) \cdot \frac{V_1}{V_2} + F_2(t)$ is a function that corrects for bleach and drift during the measurement, see Eq. 4 in Materials and Methods.

To determine $\psi(r,l)$, FEM-based (Strang & Fix, 1973) simulations of the FRAP experiment were performed. The geometry used in the simulations, specified to resemble a dividing cell, included two cylindrical compartments of length 2 μm and radius R=500 nm. A cylindrical 50 nm long septum joined the two compartments. The concentration of the fluorescent molecule (EGFP) inside the cell obeyed the three dimensional diffusion equation with a negative source term generated by photobleaching:

$$\frac{\partial c(\bar{r},t)}{\partial t} = D^* \cdot \nabla^2 c(\bar{r},t) - k_D(\bar{r},t) \cdot c(\bar{r},t) \quad \text{(S11)}$$

with boundary condition:

$$\nabla c(\bar{r},t) \cdot \hat{n} = 0 \quad \text{(S12)}$$

on the surface of the cell, with surface normal $\hat{n}$. D^* is the diffusion coefficient of EGFP, taking steric and hydrodynamic hindrance into account, which is equal to Eq. 2 in the septal region and equal to D in the compartments. The rate of degradation, $k_D(\bar{r},t)$, is only non-zero during the 40 ms of bleaching in one of the compartments, and depends on the excitation power and photophysical properties of the diffusing molecule (EGFP) (Widengren & Thyberg, 2005):

$$k_D(\bar{r},t) = \frac{\Phi_D \cdot S_1}{\tau_0} \qquad \text{(S13)}$$

Here τ_0 is the fluorescence lifetime, Φ_D the photobleaching quantum yield and S_1 the (normalized) steady state population of the (first) excited state of the EGFP. The values for τ_0, Φ_D and S_1 were specified to 2.4 ns (Jakobs *et al.*, 2000), $8 \cdot 10^{-6}$ (Peterman *et al.*, 1999) and 7%, respectively. The 7% value for S_1 was calibrated from experimental normalized fluorescence profiles immediately after the bleaching on cells lacking a visible septum. In principle it is possible to derive a value for S_1 based on the excitation intensity and various photophysical properties of EGFP. However, at intensities in the order of mW, estimations of Φ_D become uncertain. Moreover, uncertainties in $k_D(\bar{r},t)$ mainly affects the amplitude β in Eq. S10, and not significantly the other parameters, and β is always kept free in the fitting of the simulated FRAP data to Eq. S10 (see below).

Simulated FRAP relaxation curves were generated for different septal radii by solving Eq. S11 together with border condition Eq. S12 numerically. These relaxation curves were then fitted to Eq. S10. Except for $\psi(r,l)$ and β, which were allowed to vary freely, all the other parameters were in the fitting procedure fixed to the same values as used as input values in the simulations. Thereby, a relation was empirically found between *r* and $\psi(r,l)$ with *D* = 4.5 µm^2/s and with *r* varying from 6 to 500 nm (Fig. S5B). The generated relation could nicely be fitted to a sum of two exponentials (red curve in Fig. S5B):

$$\psi(r, l = 50\ nm) = \text{-}2.5797 \cdot 10^{-4} + 0.84068 \cdot e^{-r \cdot 10^9/36.1956} + 0.2745 \cdot e^{-r \cdot 10^9/222.75349} \qquad \text{(S14)}$$

Simulations were also performed to test how well Eq. S10 fits to the simulated FRAP data and how well the fitted parameters agree with the input parameters, for different sets of parameters and different cell geometries (data not shown). For cylindrical compartments, Eq. S10 was tested within the parameter range (6<r<400 nm, 3<D<6 µm^2/s, 1< L_1, L_2 <3.5 um, l = 50 nm). Eq. S10 was then found to fit well with the simulated FRAP relaxation curves and to yield fitting parameters well in perfect agreement with the input parameters.

If the compartments are ellipsoids instead of cylinders, (and their axes given by R and L1, L2) then the error in using a cylindrical model to determine *r* for ellipsoid compartments is

negligible for radii less than 20 and larger than 100 nm, but can be as high as 20% for r = 50 nm.

The influence of the septum length, l, was also tested in the same way. The correction factor $\psi(r,l)$ depends on l. However, neglecting this dependence, and assuming l to be as long as 200 nm instead of the assumed 50 nm, the estimated error will not exceed 25% for small septal radii (6 < r < 10 nm), 40% for middle sized radii (10 < r < 100 nm) and 20% for larger radii (r > 100 nm).

References

Haupts, U., S. Maiti, P. Schwille & W. W. Webb, (1998) Dynamics of fluorescence fluctuations in green fluorescent protein observed by fluorescence correlation spectroscopy. *Proc Natl Acad Sci USA* **95**: 13573-13578.

Jakobs, S., V. Subramaniam, A. Schonle, T. M. Jovin & S. W. Hell, (2000) EFGP and DsRed expressing cultures of Escherichia coli imaged by confocal, two-photon and fluorescence lifetime microscopy. *FEBS Lett* **479**: 131-135.

Peterman, E. J. G., S. Brasselet & W. E. Moerner, (1999) The fluorescence dynamics of single molecules of green fluorescent protein. *Phys Chem A* **103**: 10553-10560.

Strang, G. & G. Fix, (1973) *An analysis of the Finite Element Method*. Prentice Hall.

Widengren, J., B. Terry & R. Rigler, (1999) Protonation kinetics of GFP and FITC investigated by FCS – aspects of the use of fluorescent indicatiors for measuring pH. *Chem Phys* **249**: 259-271.

Widengren, J. & P. Thyberg, (2005) FCS cell surface measurements - photophysical limitations and consequences on molecular ensembles with heterogenic mobilities. *Cytometry A* **68**: 101-112.

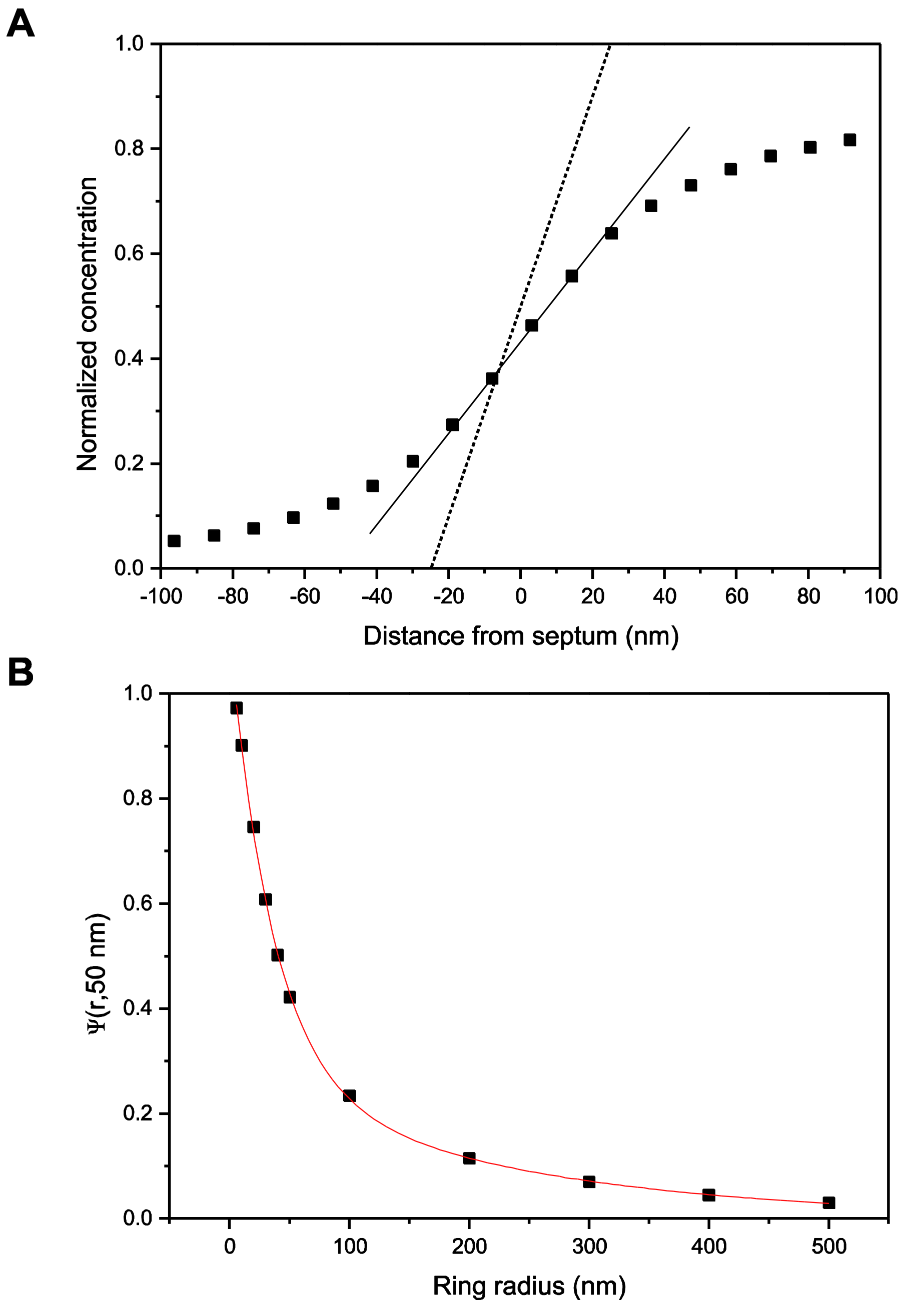

Figure S5. (A) A schematic picture of an EGFP concentration profile (black squares) over the septal ring, localized at -25 to 25 nm, with corresponding gradient $\frac{\partial c}{\partial x}$ (full line). The dashed line represents the approximate gradient $\frac{c_2 - c_1}{l}$, where c_1 and c_2 are the average normalized concentrations in each compartment. (B) The empirically derived expression (S14) for $\psi(r,l) = \frac{\partial c}{\partial x} / \frac{c_2 - c_1}{l}$ with l = 50 nm (red curve) and the corresponding simulated values (black squares).
